# Construction and validation of a clinical predictive nomogram for subacute combined degeneration of the spinal cord based on LASSO regression

**DOI:** 10.3389/fneur.2026.1939781

**Published:** 2026-09-04

**Authors:** Xiaofang Zhang, Zhiqiang Li, Dandan Liu, Wenyan Chen

**Affiliations:** 1 Department of Neurology, Yanhua Hospital, Beijing, China; 2 Department of Geriatric Medicine, Shanxi Bethune Hospital, Shanxi Academy of Medical Sciences, Third Hospital of Shanxi Medical University, Tongji Shanxi Hospital, Taiyuan, China; 3 Department of Neurology, Linfen People's Hospital, Linfen, China

**Keywords:** diagnostic efficacy, homocysteine, nomogram, prediction model, subacute combined degeneration of the spinal cord

## Abstract

**Objectives:**

This study aimed to develop and internally validate a nomogram prediction model for differentiating subacute combined degeneration of the spinal cord (SCD) from other neurological disorders, based on established clinical predictors including a symptom scale score, risk factors, and laboratory indicators.

**Methods:**

We retrospectively enrolled 80 patients with SCD (SCD group) and 80 patients with other neurological disorders (non-SCD group) treated at Beijing Yanhua Hospital from March 2015 to March 2025. Demographic characteristics, SCD-related risk factors, SCD symptom scale scores, and laboratory indicators were collected. Eight risk factors for vitamin B12 deficiency were integrated into the Risk Load Score of B12 Deficiency (RLSS). The RLSS, total SCD symptom scale score, and serum homocysteine (Hcy) were included as candidate variables. LASSO regression was used for variable compression and selection. The selected variables were entered into multivariate logistic regression to construct the nomogram prediction model. We evaluated the model’s discrimination, calibration, and clinical utility, and performed internal validation using Bootstrap resampling (1,000 Bootstrap resamples).

**Results:**

LASSO regression identified three non-zero coefficient predictors (total SCD symptom scale score, RLSS, and Hcy) from the seven candidate variables (RLSS, total SCD symptom scale score, Hcy, MCV, age, sex, and disease duration). Multivariate logistic regression showed that RLSS, Hcy, and total SCD symptom scale score were all independent predictors of SCD. The area under the curve (AUC) of the combined model was 0.954. The decrease in the C-index after Bootstrap correction was less than 0.05, indicating good calibration (Hosmer-Lemeshow *χ*^2^ = 2.326, *p* = 0.969; Brier score = 0.095). Decision curve analysis showed significant clinical net benefit across threshold probabilities of 0.1 to 0.8.

**Conclusion:**

The nomogram model based on total SCD symptom scale score, RLSS, and Hcy demonstrated good discrimination and calibration in differentiating SCD from non-SCD neurological disorders, as confirmed by internal validation. With its simple operation and readily available indicators, this model provides visualized decision support for early clinical identification of SCD. However, external validation is required before it can be recommended for routine use in primary healthcare settings.

## 1 Introduction

Subacute combined degeneration of the spinal cord (SCD) is a neurological disorder of the central and peripheral nervous systems caused by vitamin B12 (VitB12) deficiency, characterized by demyelination and axonal degeneration. It mainly affects the posterior and lateral columns of the spinal cord, with pathological features of demyelination and axonal degeneration (1). The clinical manifestations are primarily neurological, including deep sensory disturbances, ataxia, and paresthesia. In the early stage, nonspecific symptoms such as fatigue and anemia may also appear. As the disease progresses, patients may develop bowel and bladder dysfunction, cognitive decline, or even paralysis (2, 3). SCD is one of the few treatable neurological disorders, and early VitB12 supplementation can lead to significant clinical improvement or even complete recovery in the majority of patients (4, 5). However, early symptoms are often atypical and can be easily confused with cervical spondylosis, polyneuropathy, multiple sclerosis, and other conditions, making differential diagnosis challenging.

In clinical practice, a decreased serum VitB12 level is commonly used as a diagnostic criterion. Nevertheless, a considerable proportion of patients have normal serum VitB12 levels despite having functional tissue-level deficiency. Literature reports show that over 30% of SCD patients have normal VitB12 levels (6, 7); reliance on this single indicator can easily lead to missed diagnosis. Serum homocysteine (Hcy), as a sensitive metabolic marker reflecting the functional status of VitB12, typically rises earlier and more sensitively than serum VitB12 declines (8). In addition, the typical “inverted V” sign on spinal MRI has a low positivity rate, and neurophysiological examinations require specialized equipment and technicians, which limits their application in primary care settings. Given these issues, developing a comprehensive diagnostic tool that integrates clinical symptoms, easily accessible laboratory indicators, and etiological risk factors is of great clinical importance.

In this study, we retrospectively analyzed SCD cases diagnosed over the past 10 years. We integrated multiple VitB12-deficiency-related risk factors into a cumulative etiological score, combined it with the total SCD symptom scale score and serum Hcy level, and used LASSO regression for variable selection to construct a nomogram prediction model. Our aim was to develop a practical nomogram-based decision-support tool based on readily available clinical indicators and to internally validate it for the early differential diagnosis of SCD.

## 2 Materials and methods

### 2.1 Study population

This was a single-center retrospective study. Patients were divided into two groups. The SCD group included 80 patients who received a diagnosis of SCD at the Department of Neurology, Beijing Yanhua Hospital, between March 2015 and March 2025. Diagnosis followed the Chinese Consensus on the Diagnosis and Treatment of Subacute Combined Degeneration (2020) (1), with the following criteria: (1) subacute or chronic onset, with clinical signs and symptoms involving the posterior and lateral columns of the spinal cord and/or peripheral nerves; (2) serum vitamin B12 level <200 ng/L, or effective response to therapeutic vitamin B12 supplementation (the two criteria are alternative, not both required); and (3) exclusion of other spinal cord diseases. The non-SCD group included 80 patients who presented with a chief complaint of “subacute or chronic limb numbness or weakness” and were finally diagnosed with other neurological disorders, including cervical spondylosis, polyneuropathy, multiple sclerosis, and alcoholic neuropathy. Diagnoses were established by attending neurologists based on standardized clinical criteria, including characteristic clinical manifestations, appropriate imaging findings (spinal MRI for cervical spondylosis and multiple sclerosis), nerve conduction studies and electromyography for polyneuropathy, and documented history of chronic alcohol use with compatible clinical features for alcoholic neuropathy. The severity of neurological impairment in the non-SCD group was not specifically quantified, as the primary purpose of the non-SCD group was to represent the spectrum of differential diagnoses encountered in clinical practice rather than to match disease severity. Exclusion criteria were: (1) severe hepatic or renal dysfunction, heart failure, or advanced malignancy; (2) inability to cooperate with scale assessment; and (3) missing key data. Due to the retrospective design, diagnostic classification labels in the electronic medical record system had partial overlap (e.g., some peripheral neuropathies were not further subtyped). To address this, we used the principal discharge diagnosis as the primary grouping criterion: patients were assigned to the non-SCD group based on their primary diagnosis (cervical spondylosis, polyneuropathy, multiple sclerosis, or alcoholic neuropathy), regardless of any secondary diagnoses. This study was approved by the Medical Ethics Committee of Beijing Yanhua Hospital (ethics number: PHG-YHH-2025-MSWO-013), and written informed consent for participation was not required from the participants or the participants’ legal guardians/next of kin in accordance with the national legislation and institutional requirements.

### 2.2 Data collection and variable definitions

Clinical data of both groups were extracted from the electronic medical record system. The collected data included age and sex. The main clinical feature was disease duration (in months). Laboratory indicators were the most recent test results obtained after admission and before the first vitamin B12 treatment, including blood urea nitrogen (BUN), creatinine (Cr), hemoglobin (Hb), hematocrit (HCT), mean corpuscular volume (MCV), mean corpuscular hemoglobin (MCH), mean corpuscular hemoglobin concentration (MCHC), Hcy, vitamin B12, and serum folate (FA). In addition, eight binary risk factors related to SCD were collected: chronic gastritis, history of gastrectomy, anemia, long-term use of proton pump inhibitors/H2-receptor antagonists (> 3 months), alcoholism, long-term vegetarian diet, metformin use, and nitrous oxide abuse.

The SCD symptom scale, adapted from the neurologic severity scoring system described by Healton et al. (9), was used to quantify neurological symptom severity. The scale comprises seven domains: sensory disturbance, motor impairment, reflex abnormalities, autonomic symptoms (including bowel and bladder dysfunction), gait abnormalities, impaired daily function, and visual disturbance. Each domain is scored from 0 to 3 (0 = absent/normal, 1 = mild, 2 = moderate, 3 = severe), yielding a total score ranging from 0 to 20, with higher scores indicating greater severity or broader involvement. The scale was administered by attending neurologists at the time of admission based on standardized neurological examination and patient history; all assessors were board-certified neurologists with at least 5 years of clinical experience. Due to the retrospective design, formal inter-rater reliability testing was not performed; however, the scale derives from a well-established instrument with demonstrated clinical utility in cobalamin deficiency-related neurologic assessment (9). The total score was used as a continuous variable in subsequent analyses.

MRI reports of the brain, cervical cord, and thoracic cord were recorded for all patients, with particular attention to abnormal signals in the posterior columns and the typical “inverted V” sign. Neurophysiological examination results, including nerve conduction studies and somatosensory evoked potentials, were also collected to describe the ancillary test features of both groups.

### 2.3 Reorganization and pre-screening of predictors

To optimize model construction and avoid the issue of complete separation in statistical analysis, we reorganized and pre-screened the predictor variables before modeling.

First, the eight binary risk factors were integrated into a Risk Load Score of B12 Deficiency (RLSS) using an equal-weight scoring method (one point for each risk factor present). This approach was adopted for three reasons: (1) the literature has independently established all eight factors as contributors to B12 deficiency (10, 11), without robust evidence to support differential weighting; (2) a simple unweighted sum maximizes clinical usability, enabling rapid calculation at the bedside without computational tools; and (3) in our moderate-sized sample, an equal-weight approach reduces the risk of overfitting that might arise from data-driven weighting. Nevertheless, we acknowledge that this method assumes equal contributions from each factor, which may oversimplify the underlying biology; therefore, the RLSS should be interpreted as an exploratory composite score requiring external validation and potential recalibration. The RLSS was treated as a continuous variable ranging from 0 to 8 in subsequent analyses.

Second, the raw scores of the seven SCD symptom scale dimensions were summed to produce a total symptom scale score (range 0–20), with higher scores indicating greater symptom severity or more affected domains.

In the pre-screening phase prior to LASSO regression, we performed a structured variable reduction based on two principles. First, collinearity diagnostics (VIF > 10) identified two clusters of severely correlated variables: serum vitamin B12 with Hcy (both reflecting the methionine cycle), and Hb/HCT/MCH/MCHC with MCV (all representing erythrocyte morphology). To avoid unstable coefficient shrinkage, we retained only Hcy as the functional metabolic marker and MCV as the classic macrocytosis indicator, excluding their collinear counterparts. Second, based on pathophysiological reasoning, BUN, Cr, and FA were excluded *a priori* because they lack a direct mechanistic link to B12-deficiency-related SCD. This pre-filtering was not intended to bypass LASSO’s selection capability, but rather to reduce redundant noise and ensure model stability given our sample size.

After this pre-screening, the remaining non-redundant candidates—comprising RLSS, total SCD symptom scale score, Hcy, MCV, age, sex, and disease duration (totaling seven variables)—were entered into LASSO regression for final penalized selection. Among these, RLSS, total symptom score, and Hcy were considered the core pathophysiological candidates based on their direct mechanistic relevance, while MCV, age, sex, and duration were included as additional clinical covariates to allow LASSO to determine whether they provided incremental predictive value. This two-stage approach (clinically-guided pre-filtering followed by data-driven LASSO) ensures that the final model captures distinct biological dimensions while maintaining computational stability and interpretability.

### 2.4 Statistical analysis

Baseline data were analyzed using SPSS 26.0. Continuous variables with a normal distribution were expressed as mean ± standard deviation (SD), and comparisons between two groups were made using the independent samples *t*-test. Non-normally distributed data were expressed as median (Q1, Q3), and group comparisons were made using the Mann–Whitney U test. Categorical data were expressed as frequencies (%), and group comparisons were made using the chi-square test or Fisher’s exact test. Normality was assessed using the Shapiro–Wilk test. Prediction model construction and validation were performed using R 4.3.3. The following R packages were utilized: glmnet (version 4.1–8) for LASSO regression, rms (version 6.8–2) for nomogram construction and model calibration, pROC (version 1.18.5) for ROC curve analysis and AUC comparisons using the DeLong test, and rmda (version 1.6) for decision curve analysis. For LASSO regression, the optimal penalty parameter *λ* was selected using 10-fold cross-validation with the λ.min criterion, which corresponds to the *λ* value that minimizes the cross-validated mean squared error. The 1-standard-error rule (λ.1se) was also examined but yielded a more parsimonious model with inferior AUC; therefore, we adopted λ.min to balance predictive performance and model complexity. The variables selected by LASSO were then entered into multivariate logistic regression to calculate odds ratios (OR) and their 95% confidence intervals (CI). Based on the multivariate regression results, a nomogram prediction model was constructed using the rms package. Model performance was evaluated as follows. Discrimination was assessed using the receiver operating characteristic (ROC) curve and the area under the curve (AUC), with group comparisons performed using the DeLong test. Calibration was evaluated using calibration curves combined with the Hosmer-Lemeshow goodness-of-fit test and Brier score. Clinical net benefit was evaluated using decision curve analysis (DCA). Internal validation was planned using the Bootstrap resampling method (1,000 Bootstrap resamples) to calculate the optimism-corrected C-index. All tests were two-sided, and a *p* value < 0.05 was considered statistically significant.

## 3 Results

### 3.1 Baseline clinical characteristics

During the study period, 213 consecutive patients with subacute or chronic limb numbness or weakness were initially identified from the electronic medical record system. Of these, 53 patients were excluded: 28 had severe hepatic or renal dysfunction, heart failure, or advanced malignancy; 12 were unable to cooperate with the scale assessment; and 13 had missing key data. The remaining 160 patients were included in the final analysis, comprising 80 patients with confirmed SCD and 80 patients with other neurological disorders, including cervical spondylosis, polyneuropathy, multiple sclerosis, and alcoholic neuropathy. Due to the retrospective nature of the data, we have provided a qualitative description of the control group composition rather than reporting exact counts for each diagnostic subtype. There were no significant differences between the two groups in age or sex distribution (both *p* > 0.05). Compared with the non-SCD group, the SCD group had a significantly longer disease duration and a higher RLSS (both *p* < 0.01). Among the eight RLSS components, chronic gastritis, anemia, and long-term PPI/H2RA use were significantly more frequent in the SCD group, whereas metformin use was more common in the non-SCD group, and alcoholism showed comparable frequencies between the two groups. Regarding laboratory indicators, the SCD group had lower hemoglobin and hematocrit levels (*p* < 0.01) and a larger mean corpuscular volume (*p* < 0.05) than the non-SCD group. Serum homocysteine (Hcy) was significantly higher, while serum vitamin B12 was lower in the SCD group. The total SCD symptom scale score was also higher in the SCD group (*p* < 0.05) (Table 1). In terms of ancillary examinations, among the 80 SCD patients, 20 (25%) showed abnormal signals on spinal MRI, with 8 (10%) presenting the typical “inverted V” sign in the posterior columns. Regarding neurophysiological findings, 64 patients (80%) had abnormal somatosensory evoked potentials in both lower limbs, and 58 patients (72.5%) showed peripheral nerve damage of the common peroneal nerve.

**Table 1:** Comparison of clinical characteristics between the SCD and non-SCD groups.

Variable	SCD group (n = 80)	Non-SCD group (*n* = 80)	Statistic	*p*-value
Age (years, mean ± SD)	66.95 ± 8.65	64.45 ± 12.27	*t* = 0.745	0.462
Male [*n* (%)]	44 (55.0%)	52 (65.0%)	*χ*^2^ = 1.667	0.197
Disease duration [months, median (Q1, Q3)]	5.00 (2.00, 24.00)	0.75 (0.25, 1.50)	*Z* = −3.757	<0.001
RLSS [median (Q1, Q3)]	1.50 (1.00, 3.00)	1.00 (0.00, 1.00)	*Z* = 2.854	0.006
Blood urea nitrogen [mmol/L, mean ± SD]	5.05 ± 1.82	5.58 ± 2.21	*t* = −0.832	0.411
Creatinine [μmol/L, median (Q1, Q3)]	64.05 (59.60, 81.58)	72.15 (60.15, 89.23)	*Z* = 1.028	0.314
Hemoglobin [g/L, median (Q1, Q3)]	127.0 (107.75, 136.5)	139.5 (131.25, 149.0)	*Z* = 2.773	0.005
Hematocrit (%, mean ± SD)	34.61 ± 7.55	41.47 ± 5.37	*t* = −3.313	0.002
MCV (fL, mean ± SD)	103.80 ± 12.62	96.49 ± 6.58	*t* = 2.298	0.029
MCH [pg, median (Q1, Q3)]	34.85 (31.90, 39.43)	31.65 (30.38, 33.28)	*Z* = −2.408	0.015
MCHC (g/L, mean ± SD)	343.15 ± 13.81	333.90 ± 14.51	*t* = 2.065	0.046
Hcy [μmol/L, median (Q1, Q3)]	46.59 (13.85, 84.96)	13.26 (10.75, 15.47)	*Z* = 3.449	<0.001
Folate [ng/L, median (Q1, Q3)]	6.30 (2.75, 15.58)	13.30 (8.80, 16.78)	*Z* = 2.218	0.026
Vitamin B12 [ng/L, median (Q1, Q3)]	221.05 (190.05, 352.35)	313.55 (243.15, 420.30)	*Z* = 2.083	0.038
SCD symptom scale score [points, median (Q1, Q3)]	6.00 (4.25, 9.00)	0.00 (0.00, 3.00)	*Z* = 3.888	<0.001

SD, standard deviation; RLSS, risk load score of B12 deficiency; MCV, mean corpuscular volume; MCH, mean corpuscular hemoglobin; MCHC, mean corpuscular hemoglobin concentration; Hcy, homocysteine.

### 3.2 LASSO regression analysis of factors associated with SCD

**Figure 1:**
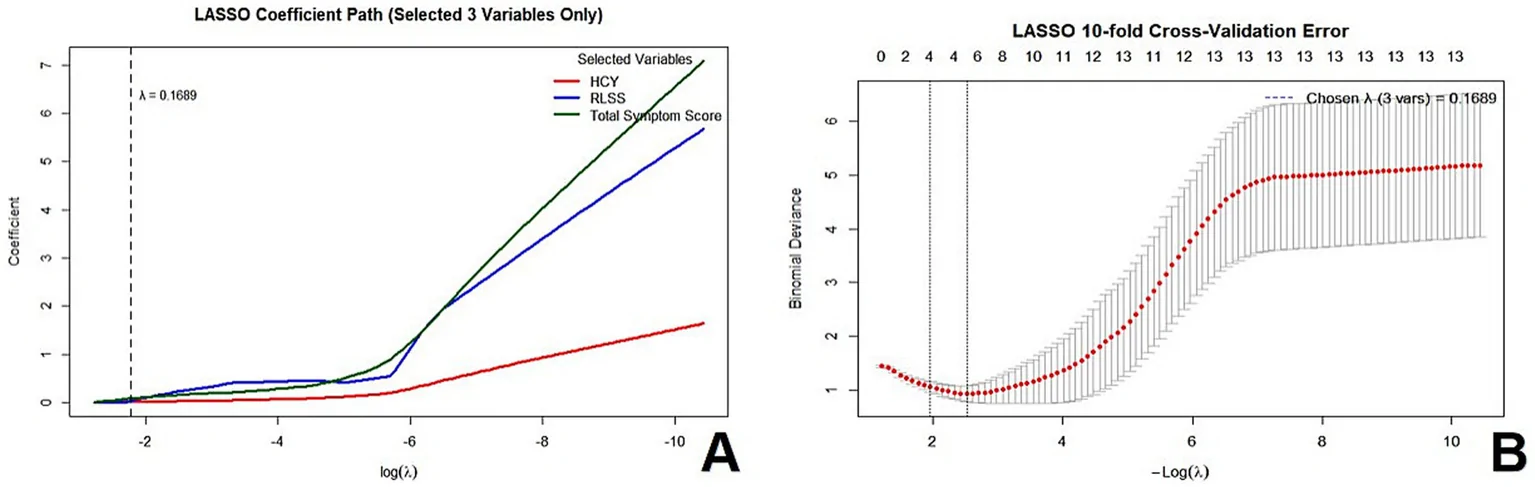
Results of LASSO regression variable selection. **(A)** LASSO regression coefficient path diagram. **(B)** 10-fold cross-validation mean squared error plot.

All candidate variables were entered into LASSO regression, and the optimal penalty coefficient *λ* was determined by 10-fold cross-validation (λ.min criterion). Figure 1A shows the trajectory of regression coefficients for each variable as *λ* increases. Figure 1B shows the cross-validation mean squared error plot. At λ.min, only three variables retained non-zero regression coefficients (total SCD symptom scale score, RLSS, and Hcy), while the other four variables (MCV, age, sex, and disease duration) were compressed to zero. The final selected predictors were: total SCD symptom scale score (coefficient = 0.0822), RLSS (coefficient = 0.0302), and Hcy (coefficient = 0.0135; Table 2).

**Table 2:** Predictors selected by LASSO regression and their coefficients.

Variable	LASSO coefficient	Rank
Total SCD symptom scale score	0.082	1
RLSS	0.030	2
Hcy	0.014	3

LASSO regression used 10-fold cross-validation with the λ.min criterion. RLSS ranges from 0 to 8 points. A larger absolute coefficient indicates a stronger predictive contribution.

### 3.3 Multivariate logistic regression analysis

**Figure 2:**
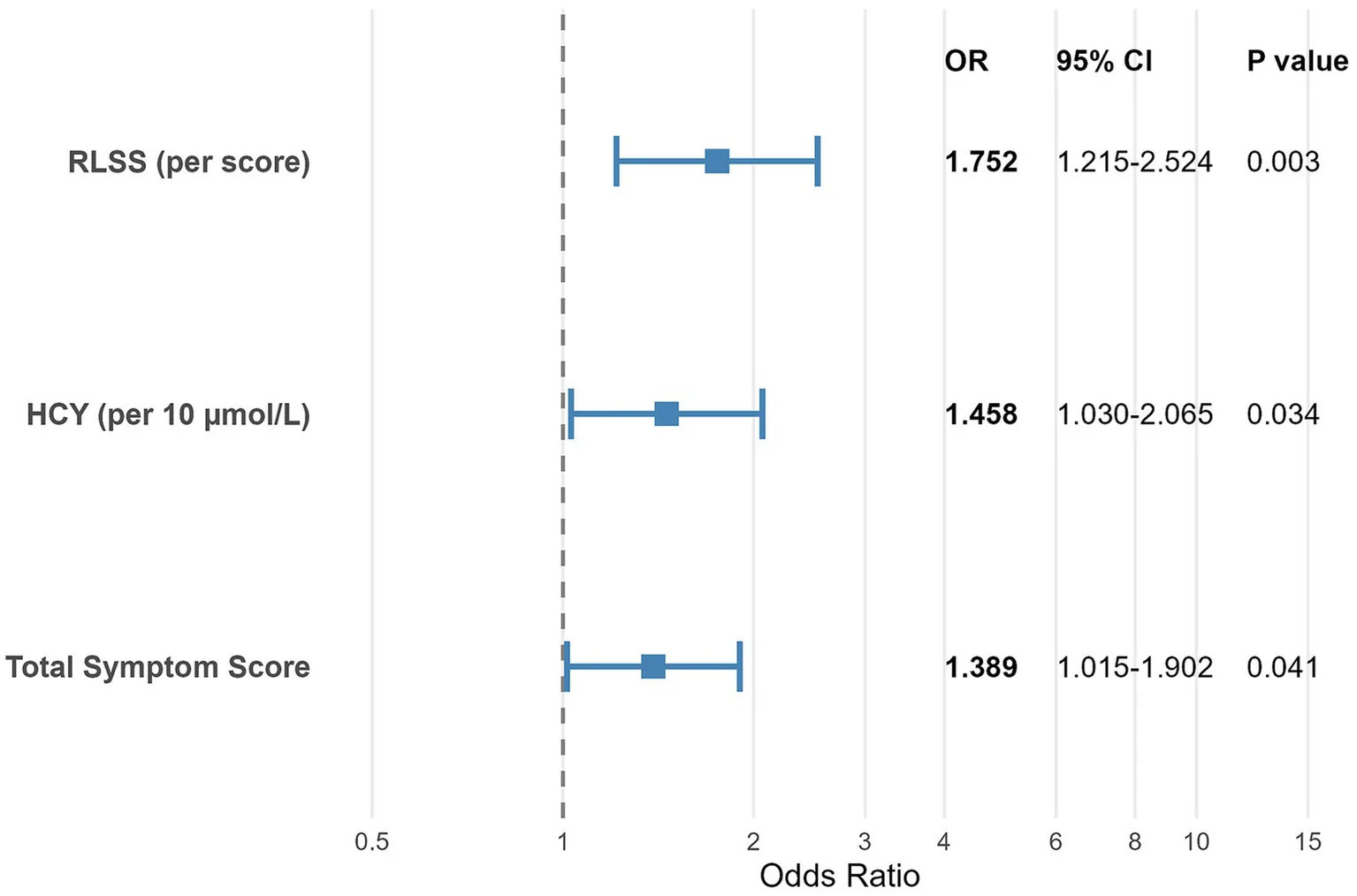
Forest plot of multivariate logistic regression.

The three predictors selected by LASSO (total SCD symptom scale score, RLSS, and Hcy) were entered into a standard multivariate logistic regression model. Hcy was entered per 10 μmol/L, while RLSS and the total SCD symptom scale score were entered as raw scores (per 1-point increase). The results (Table 3; Figure 2) showed that all three variables were independent predictors of SCD (all *p* < 0.05). For each 1-point increase in RLSS, the risk of SCD increased by 75.2% (OR = 1.752, 95% CI: 1.215 to 2.524, *p* = 0.003). For each 10 μmol/L increase in Hcy, the risk of SCD increased by 45.8% (OR = 1.458, 95% CI: 1.030 to 2.065, *p* = 0.034). For each 1-point increase in the total SCD symptom scale score, the risk of SCD increased by 38.9% (OR = 1.389, 95% CI: 1.015 to 1.902, *p* = 0.041). To facilitate independent application and external validation, the complete logistic regression equation is available from the corresponding author upon reasonable request. The regression coefficients for RLSS, Hcy (per 10 μmol/L), and total SCD symptom score are 0.561, 0.377, and 0.329, respectively, with an intercept of approximately −2.816. These coefficients can be used to compute individual predicted probabilities using the standard logistic transformation *p* = 1/[1 + exp.(–LP)], where LP = −2.816 + 0.561 × (RLSS) + 0.377 × (Hcy/10) + 0.329 × (total symptom score).

**Table 3:** Multivariate logistic regression analysis of factors associated with SCD.

Variable	OR	95% CI	*p*-value
RLSS (per 1 score)	1.752	1.215–2.524	0.003
Hcy (per 10 μmol/L)	1.458	1.030–2.065	0.034
Total SCD symptom scale score (per 1 score)	1.389	1.015–1.902	0.041

RLSS ranges from 0 to 8 points; total SCD symptom scale score ranges from 0 to 20 points.

### 3.4 Construction of the nomogram prediction model for SCD diagnosis

**Figure 3:**
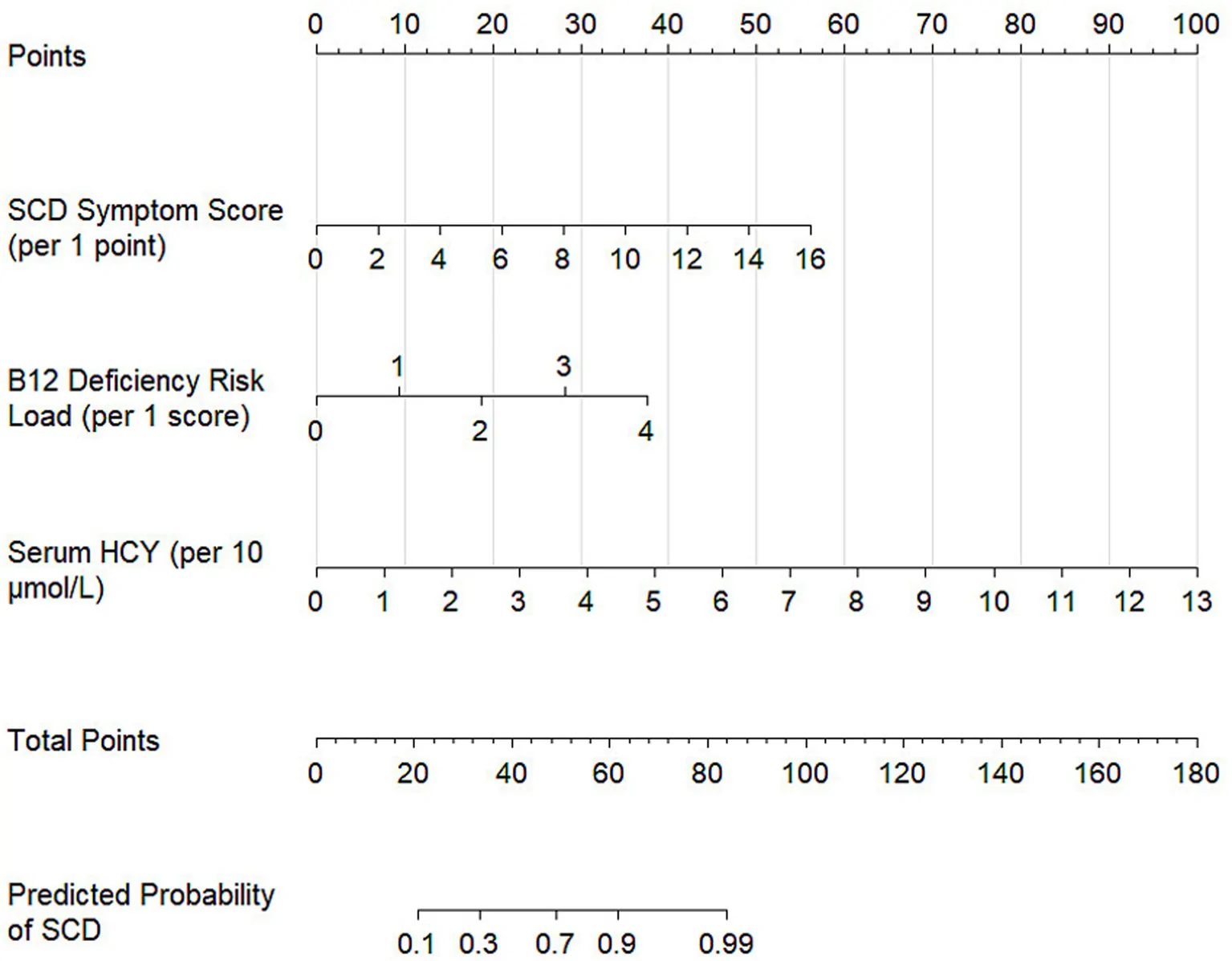
Nomogram for predicting SCD risk (RLSS ranges from 0 to 8 in the original definition; however, the axis displayed in the figure reflects the observed range (0–4) in the current study population. Predictions for RLSS values above 4 have not been empirically validated and should be interpreted with caution).

Based on the multivariate logistic regression results, the three independent predictors (total SCD symptom scale score, RLSS, and Hcy) were incorporated into the final prediction model to construct a visual nomogram (Figure 3). The nomogram converts the regression coefficient of each predictor into a score scale of 0 to 100 points. Users can obtain the individual score for each indicator by projecting the patient’s actual value onto the Points scale. After summing these scores to obtain the Total Points, the predicted probability of SCD can be obtained by projecting downward onto the “Predicted Probability of SCD” axis.

### 3.5 Performance analysis of the nomogram model

**Figure 4:**
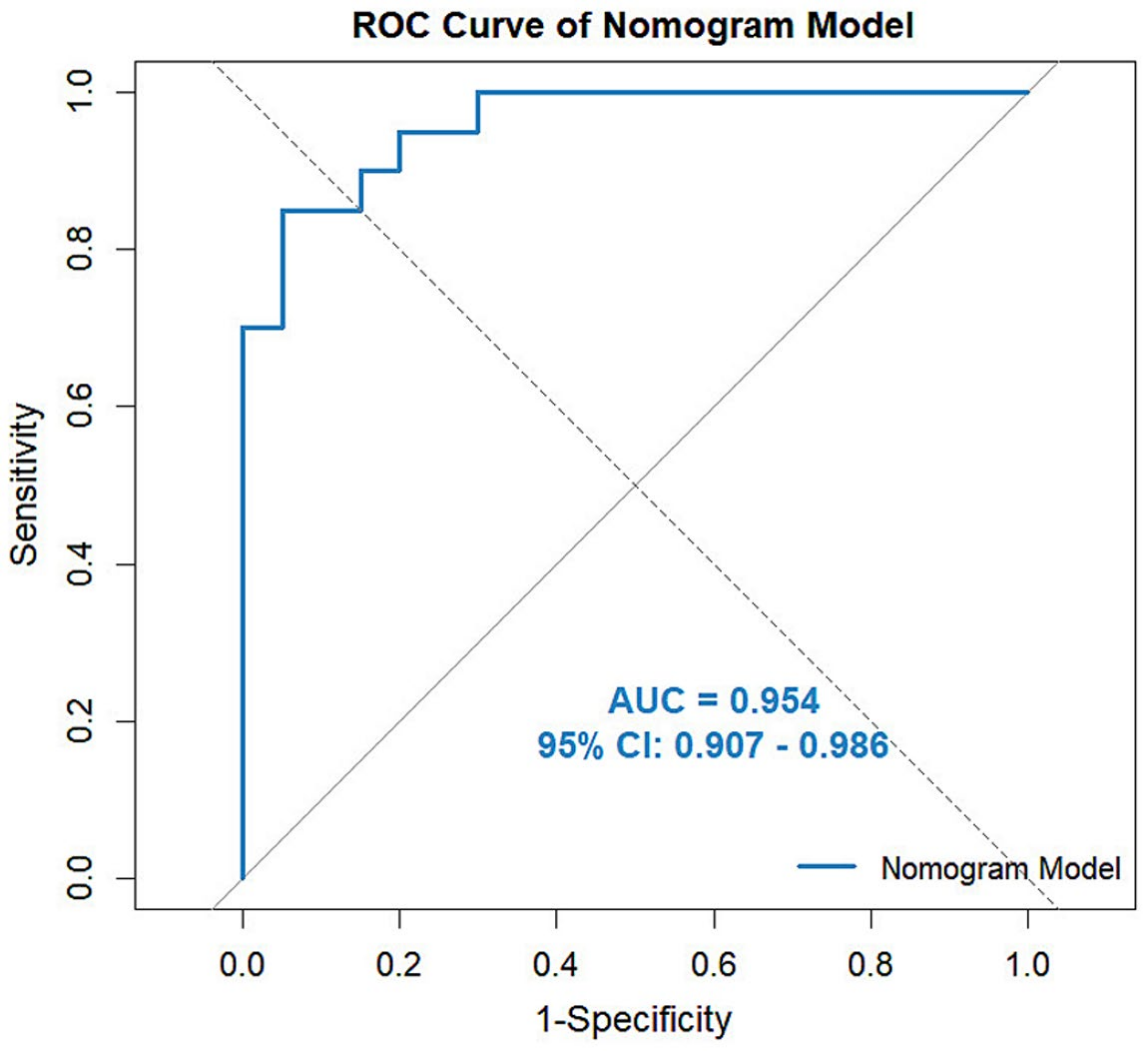
ROC curve of the nomogram for diagnosing SCD.

ROC curves were used to evaluate the discriminatory performance of the nomogram model and each individual predictor (with the SCD group as positive cases). The results (Table 4; Figure 4) showed that the AUC of the combined nomogram model was 0.954 (95% CI: 0.907–0.986), which was significantly higher than that of each individual indicator (DeLong test, all *p* < 0.05). The AUCs for the total SCD symptom scale score, RLSS, and Hcy were 0.825 (95% CI: 0.731–0.958), 0.783 (95% CI: 0.604–0.896), and 0.795 (95% CI: 0.674–0.964), respectively. The C-index of the combined model was 0.954.

**Table 4:** Diagnostic performance of individual predictors and the combined model for SCD.

Variable	AUC	95% CI	Sensitivity	Specificity
Total SCD symptom scale score	0.825	0.731–0.958	0.812	0.738
RLSS	0.783	0.604–0.896	0.750	0.725
Hcy (per 10 μmol/L)	0.795	0.674–0.964	0.725	0.763
Nomogram model	0.954	0.907–0.986	0.875	0.912

### 3.6 Model calibration and internal validation

**Figure 5:**
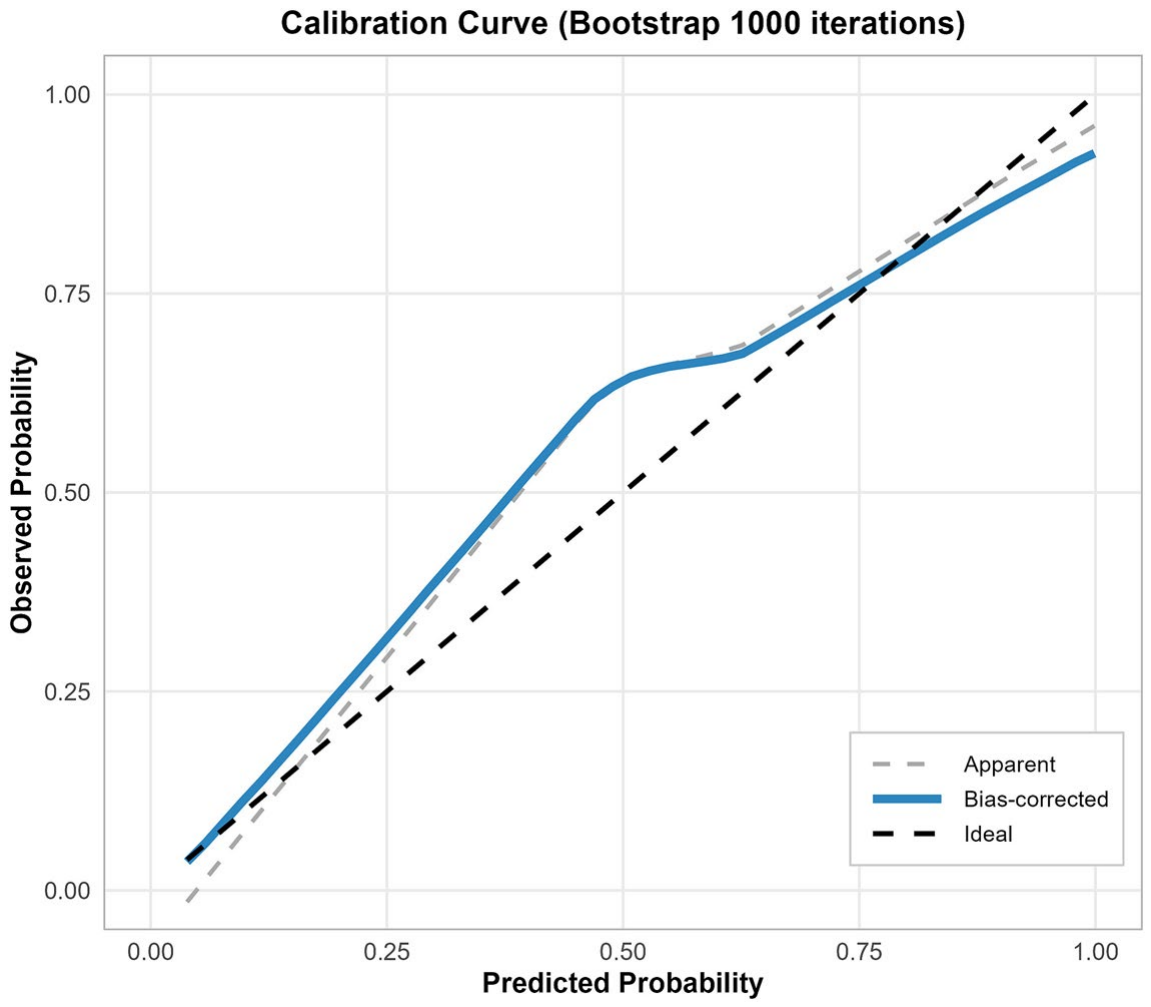
Calibration curve of the model (bootstrap 1,000 resamples).

Internal validation was performed using Bootstrap resampling (1,000 resamples). The calibration curve (Figure 5) showed that the bias-corrected curve closely approximated the ideal 45° diagonal line across the full probability range, indicating good agreement between predicted and observed probabilities. The mean absolute error of the calibration curve was 0.065, meaning that the average absolute deviation between predicted and observed probabilities was approximately 6.5%. The Hosmer-Lemeshow goodness-of-fit test yielded *χ*^2^ = 2.326 (degrees of freedom = 8), *p* = 0.969, indicating no significant difference between predicted and observed probabilities. The Brier score was 0.095, below the acceptable threshold of 0.25, reflecting good accuracy of the model’s probability predictions. For discrimination, the Bootstrap validation showed that the original C-index was 0.954, and the optimism-corrected C-index was 0.921 (decrease < 0.05), indicating no significant overfitting and good potential for generalizability.

### 3.7 Decision curve analysis

**Figure 6:**
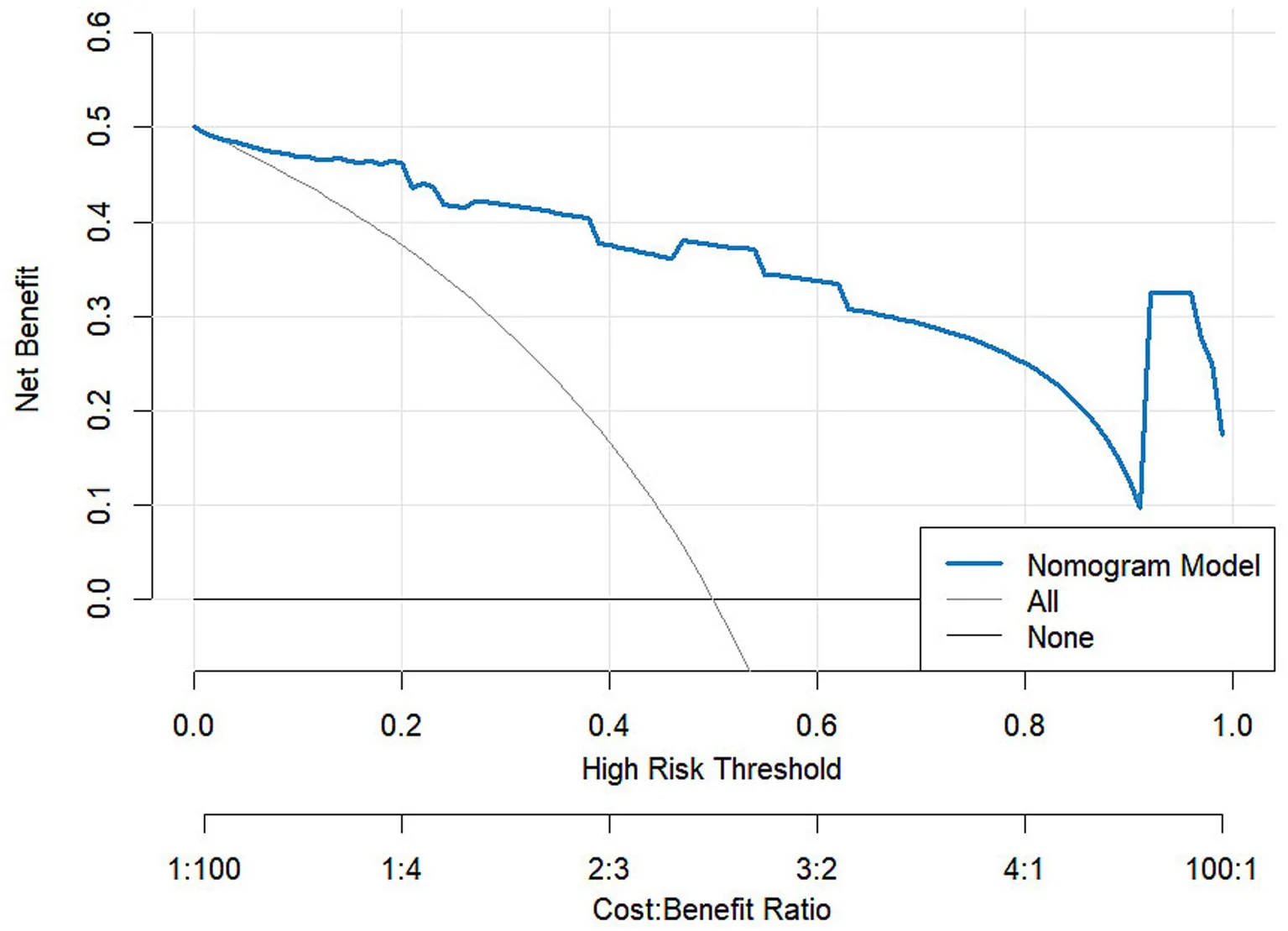
Decision curve analysis (DCA).

DCA was used to evaluate the clinical net benefit of the model (Figure 6). The results showed that within the threshold probability range of 0.1 to 0.8, the nomogram model provided a higher clinical net benefit than both the “treat all” and “treat none” strategies. The net benefit advantage was most pronounced within the threshold probability range of 0.2 to 0.7. When the threshold probability was below 0.1 or above 0.8, the net benefit of the model approached that of the extreme strategies. These findings indicate that the nomogram model provides good net benefit within the clinically applicable threshold probability range of 0.1 to 0.8 and can offer effective quantitative decision support for the clinical differential diagnosis of SCD.

## 4 Discussion

In this study, we identified three core predictors—total SCD symptom scale score, RLSS, and Hcy—using LASSO regression and constructed a clinical prediction nomogram for SCD. After internal validation with 1,000 Bootstrap resamples, the model showed good discrimination (AUC = 0.954, 95% CI: 0.907 to 0.986), good calibration (Hosmer-Lemeshow test *p* = 0.969, Brier score = 0.095), and favorable clinical net benefit. This model provides a simple, visualized, and quantitative tool for the early differential diagnosis of SCD. It achieves high diagnostic performance using only three easily obtainable clinical indicators, without the need for MRI or neurophysiological equipment. This suggests potential applicability in primary healthcare settings, pending external validation.

From a pathophysiological perspective, the three predictors identified in this study cover the complete logical chain of “etiology – metabolism – clinical manifestation” in the development of SCD. RLSS reflects the cumulative burden of upstream etiological exposure—factors such as chronic gastritis, gastrectomy, long-term proton pump inhibitor use, alcoholism, and vegetarian diets interfere with the absorption, activation, or utilization of vitamin B12, thereby providing the pathophysiological basis for SCD (10). Hcy, as a key metabolic intermediate in the vitamin B12-dependent methionine cycle, directly reflects the metabolic consequences of functional vitamin B12 deficiency at the tissue level. The total SCD symptom scale score provides a systematic quantification of the extent and severity of downstream neurological involvement. These three factors contribute to the prediction model in a complementary rather than simply additive manner, which likely explains the high AUC of 0.954 achieved by the combined model.

Serum Hcy showed strong independent predictive value in this study, while routine serum vitamin B12 was excluded during the variable pre-screening stage. We emphasize that this exclusion does not, in any way, negate the central etiological role of vitamin B12 in SCD pathogenesis, but rather reflects a methodological and clinical necessity driven by two considerations. First, from a statistical perspective, serum B12 and Hcy exhibited severe multicollinearity (VIF > 10), as they are metabolically intertwined in the methionine cycle. Retaining both in logistic regression with our sample size would lead to unstable coefficient estimates and inflated variances, compromising model reliability. Second, and more importantly, from a pathophysiological and clinical standpoint, these two biomarkers measure fundamentally different constructs. Serum B12 quantifies the total circulating pool bound to haptocorrin, representing an inactive storage reservoir, whereas Hcy directly reflects intracellular B12-dependent methionine synthase activity. In tissue-level B12 deficiency, impaired synthase activity leads to Hcy accumulation often before serum B12 levels fall below the normal range. Thus, Hcy serves as an early sentinel of functional deficiency, while serum B12 is a relatively late indicator of depleted stores. Selecting Hcy over B12 was therefore a deliberate choice to prioritize a functional, dynamic biomarker over a static storage marker, aiming to enhance diagnostic sensitivity for early-stage SCD.

This choice is further supported by the well-recognized clinical phenomenon that a considerable proportion of patients with tissue-level B12 deficiency have normal serum B12 levels. Cao et al. (7) reported that over 30% of SCD patients have serum B12 within the normal range, indicating that reliance on this single measure is insufficient for diagnosis. In our study, the median Hcy level in the SCD group was 46.6 μmol/L—3.5 times higher than that in the non-SCD group—further confirming the strong sensitivity of this indicator in reflecting SCD-related metabolic disturbances. For patients with high clinical suspicion but normal B12 levels, elevated Hcy should prompt timely B12 supplementation without waiting for B12 to decline, aligning with current functional-deficiency diagnostic paradigms (12). We acknowledge that holotranscobalamin (active B12) was not measured, as it is not yet routinely available in most primary care settings, and that folate status may influence Hcy levels since Hcy metabolism also depends on folate as a methyl donor. Thus, elevated Hcy is not entirely specific for B12 deficiency, and folate deficiency could confound its interpretation as a B12-specific marker. Future studies incorporating holotranscobalamin and folate measurements alongside Hcy may further refine the predictive model and improve the specificity of metabolic assessment.

RLSS entered the final model with the largest effect size, confirming the role of cumulative burden of B12-deficiency risk factors in the pathogenesis of SCD. In clinical practice, SCD is often not caused by a single risk factor, but rather results from the long-term accumulation of multiple factors. Most previous studies have used simple lists or counts of individual risk factors. In contrast, the RLSS developed in this study integrates eight risk factors into a continuous variable using an equal-weight scoring method, transforming scattered information into a standardized, quantifiable composite score. Clinicians can complete the assessment within minutes, greatly improving the efficiency and operability of etiological evaluation. In addition, RLSS effectively avoids the issue of complete separation caused by zero events of certain individual risk factors in the non-SCD group, ensuring stable convergence of the logistic regression model. In our study population, cases with a history of gastrectomy or nitrous oxide abuse were extremely rare (11). If these factors had been entered into the regression model as independent variables, their coefficient estimates would have been highly unstable or even unable to converge. However, within the RLSS framework, they still contribute to the overall etiological burden through cumulative scoring, preserving all etiological information while ensuring computational stability. The OR value of RLSS was higher than that of Hcy and the total symptom scale score, suggesting that the cumulative effect of etiological burden may have the strongest discriminative power in predicting SCD risk. When evaluating suspected patients, clinicians should systematically review vitamin B12-deficiency-related risk factors rather than focusing solely on a single factor or laboratory indicator.

The independent predictive value of the total SCD symptom scale score was also validated. This scale provides a systematic and quantitative assessment of the common but scattered neurological symptoms of SCD (9), indicating that the cumulative burden of neurological signs makes an independent and stable contribution to differential diagnosis. In our study, the median total scale score was 6.00 in the SCD group versus 0.00 in the non-SCD group (*p* < 0.001). This large difference between the two groups reflects the scale’s good discriminative potential. The scale is based on a standard neurological physical examination and requires no equipment, making it highly clinically feasible. For patients with high scale scores, even if serum vitamin B12 levels are normal, further Hcy testing and detailed etiological evaluation should be performed. This approach is highly consistent with the principle recommended by the 2024 UK National Institute for Health and Care Excellence (NICE) guideline, which suggests screening for vitamin B12 deficiency in individuals with at least one common symptom and one risk factor (13). Beyond aiding diagnosis, the scale provides a standardized neurological assessment framework that helps avoid missed diagnoses due to non-systematic history taking, which is particularly important in primary healthcare settings and for junior physicians.

Regarding diagnostic methods, the positivity rate of spinal MRI in our study was only 25%, and only 10% showed the typical “inverted V” sign—lower than the 40–70% reported in the literature (14). This may be related to the relatively short disease duration in our patients (median 5 months). The T2-weighted hyperintensity in the posterior columns on MRI reflects morphological changes that occur after tissue edema and demyelination reach a certain threshold. In the early stage of SCD, metabolic and functional disturbances predominate, and significant imaging changes have not yet developed, which limits the sensitivity of MRI in early diagnosis (14, 15). Neurophysiological examinations, although more sensitive (abnormal somatosensory evoked potentials in both lower limbs were found in 80% of our SCD patients), require specialized technicians and equipment. Interpretation of results also requires experience, making routine use difficult in primary settings. Moreover, the abnormalities are often non-specific, offering limited value in differential diagnosis. In contrast, our model requires only three indicators: symptom scale score (standard physical examination), RLSS (detailed history taking), and serum Hcy (single blood sample). These three indicators are accessible in primary hospitals. With an AUC of 0.954 and simple, rapid operation, the model may represent a practical alternative for early SCD identification in resource-limited settings, pending external validation.

This study has several limitations. First, the single-center retrospective design with equal-size sampling does not reflect true SCD prevalence and may overestimate diagnostic performance. Moreover, all SCD patients were hospitalized with relatively severe or established diagnoses, leaving the model’s utility in outpatient, early-stage, or atypical cases unclear. Most importantly, the lack of external validation precludes clinical implementation, as internal Bootstrap validation cannot substitute for independent assessment in populations with different case-mix and prevalence. Additionally, the 10-year recruitment period may have introduced temporal heterogeneity in diagnostic criteria and laboratory assays. The sample size was determined by available cases rather than formal calculation, though the EPV supports model feasibility. Additionally, due to overlapping diagnostic labels in the electronic medical record system, we were unable to provide exact counts for each non-SCD diagnostic subtype. This limitation should be considered when interpreting the composition of the control group. Second, the RLSS and SCD symptom scale are newly developed/adapted tools requiring further validation. The retrospective, unblinded design may have introduced measurement bias, particularly for symptom scale assessments, and precluded assessment of inter-rater reliability or determination of an optimal diagnostic threshold. While their validity is supported by significant independent associations with SCD, the RLSS uses an unweighted cumulative score without external calibration, despite the eight risk factors not contributing equally to B12 deficiency risk. Both instruments require external validation and prospective evaluation. Third, we did not measure more specific metabolic markers such as MMA or active B12, though these are not routinely available in primary care. Nitrous oxide-related SCD was rare in our center, limiting model applicability to such cases. Future studies should externally validate the model and explore subtype-specific tools.

## 5 Conclusion

In conclusion, the SCD nomogram prediction model constructed in this study is based on the pathophysiological chain of cumulative etiology, metabolic disturbance, and clinical manifestations. The model integrates three predictors that are easy to obtain. Internal validation demonstrated good discrimination, calibration, and clinical net benefit. Its main advantage is that it does not rely solely on serum vitamin B12 or require specialized equipment such as MRI or electrophysiology. This provides an objective, quantitative, and visual decision-making tool for early SCD identification, with potential applicability in resource-limited settings pending external validation.

## Data Availability

The original contributions presented in the study are included in the article/supplementary material, further inquiries can be directed to the corresponding author.
